# Miscellanea

**Published:** 1855-09

**Authors:** 


### MISCELLANEA.

### BURIAL REGULATIONS IN ENGLAND.

Fifty-eight Burial Boards in the United Kingdom have issued certain rules with regard to burials and burial fees. The following have an important social and sanitary bearing.

At Beccles, no more than one body shall be buried in any vault or grave, except in a vault or grave purchased for the exclusive use of a family. No interment shall take place in any vault or grave, not filled up with earth, unless the corpse be enclosed in an air-tight metallic coffin. When more than one body is interred in any family grave, a layer of earth 18 inches in thickness at least shall be left between each coffin. Wherever a burial has taken place, except in a private vault, the surface of the grave shall be forthwith covered with fresh turf, or planted with shrubs, etc., unless a monument has to be erected; and in no case shall the bare earth be left exposed.

At Bungay, in addition to rules of a similar kind, it is agreed that no grave or vault less than 5 feet, nor more than 10 feet in depth from the surface will be allowed, and in every case the top of a coffin must be at least four feet from the surface of the ground. Burial will not be allowed in the chapels, nor within 10 feet from the walls thereof, nor within 5 feet from the fences.

At Chippenham, to similar regulations, it is added that every grave or vault shall be not less than 10 feet in depth; and none but bricked graves or vaults, purchased for the exclusive use of a family, are to be re-opened. No coffin, other than that of wood, is allowed in any grave but a bricked grave; and not in a bricked grave unless purchased for the use of a family.

In Dover, it is likewise ruled that no vaults or brick graves shall be opened from the top, unless the ground required for entrance shall have been purchased.

In Falmouth it is ordered, that the grave space set apart for each person, whether adult or not (except in the case of still-born children,) shall be 9 feet long and 4 feet 6 inches wide, and shall extend to the depth of 8 feet from the surface; and that with the exception of purchased vaults and private graves, interments shall take place in every grave consecutively, no grave being re-opened for another interment until every grave space in the burial ground shall have been buried in, or appropriated; nor even then unless upwards of fourteen years shall have elapsed since the last burial therein; and if, on re-opening any grave the corpse last interred therein shall be found undecayed, the remains shall not be disturbed, but such grave shall be filled up again with earth, and not used until the complete decay of such corpse. In the case of purchased graves, if more than one body is to be buried, notice thereof must be given to the clerk of the Board at the time of bespeaking the grave, which shall be dug to such a depth beyond 8 feet, as will admit a layer of earth 18 inches thick, at least, between each coffin; and on the soil over every coffin so deposited, shall be placed a coffin-shaped slate or flagstone, as a preventive to the gravedigger from going deeper when excavating for any future interment, and the depth of such grave shall be recorded in the register. In the case of purchased vaults, the same notice must be given, and the excavation carried to a depth of 2 feet 6 inches, at least, beyond 8 feet, for every body more than two to be therein interred, and each body shall be enclosed in an air-tight metallic coffin, and be separately entombed, the enclosing masonry being hermetically cemented; and no coffin shall be deposited in any such vault or brick grave nearer the surface than 4 feet, measuring from the upper surface of the arch or covering stone to the level of the ground, and the depth of every such grave shall be recorded in the register.

With respect to the rate of burial fees so much difference exists in various places, that no correct general idea can be given with regard to them. In one instance, the officiating minister's fee is two shillings, in another not less than three guineas.

---

### WORKHOUSE ACCOMMODATION IN ENGLAND, &c.

The workhouse accommodation in England, to a population of 16,826,492, is 204,633; in Wales, population 1,000,975, it is 6,936; in Scotland, population 2,888,742, is 9779, about to be increased however, and in Ireland, population 6,552,055, is 214,281; that is for about 1 in 82 in England, 1 in 144 in Wales, 1 in 295 in Scotland, and 1 in 31 in Ireland. From an analysis of the proportion of workhouse accommodation to the population of the various counties of England, some curious results are obtained. Thus, we find the largest relative accommodation in Suffolk, Sussex, and Wilts, the proportion being about 1 in 48. That of Berks and Kent, however, is nearly as large. Durham presents the least accommodation, viz., about 1 in 204. The West Riding of Yorkshire, Monmouth, Northumberland, Chester, Derby, Worcester, Lancaster, Warwick, Hereford, and Stafford also have but little workhouse accommodation, the ratio increasing in the order in which the places are named. In Middlesex the ratio is 1 in 75.

A large number of parishes in Scotland accommodate their poor in the poor-houses of other parishes.

### WORKHOUSE RETURNS, LONDON.

There are in London and its environs thirty-eight unions, some of which have two or more establishments, the total number taken together being forty-nine. Of these forty-nine, forty-one may be said to have separate wards for infectious disorders; three have not, and the remainder for the most part remove to hospitals patients of such character. The number of inmates in each house ranges from 1761 to 115, the average being about 512. The number of patients under medical treatment during 1854 averaged about 2345(?); the number of medical men employed at each workhouse being mostly 1; and of paid nurses, 153(?), in thirty-one workhouses. The St. Marylebone Union has the greatest number of inmates, viz., 1761; and the Petty France establishment of the united parishes of St. Margaret and St. John, Westminster, the least, viz., 115; the average being 512. The patients under medical treatment at each workhouse during 1854 average 1078; the medical men employed being mostly one to each workhouse; and the average of paid nurses about three.

### MALT CONSUMED IN THE UNITED KINGDOM.

In the year ending the 5th July, 1853, there were made in England, 36,076,370 bushels; in Scotland, 4,244,744 bushels; and in Ireland, 1,594,427 bushels. In the year ending July 5, 1854, in England, 34,003,266 bushels; in Scotland, 3,785,728 bushels; and in Ireland, 3,268,101 bushels. In the year ending July 1, 1855, in England, 31,660,285 bushels; in Scotland, 3,268,101 bushels; and in Ireland, 1,470,282 bushels. The totals in each of the above named years are 41,915,541; 39,511,220; and 36,398,668 bushels respectively.

### Use of SPIRITS OF WINE in Arts and Manufactures.

By a parliamentary return from Professors Graham, Hoffman and Redwood, we find that the principal uses to which spirit of wine is, or may be applied, independently of its use as a beverage, appear to be the following. As a solvent of resinous substances which, when thus dissolved, are used in the manufacture of hats, and otherwise as varnishes. As a solvent employed in the manufacture of many chemical preparations, including the alkaloids and other organic products, which are principally used in medicine. For the production of ether, chloroform, sweet spirit of nitre, and fulminating mercury. For burning in spirit-lamps as a source of heat, and for mixing with oil of turpentine, or other hydrocarbons, for burning in lamps as a source of light. As a solvent and menstruum for administering the active constituents of animal and vegetable substances used in medicine in the form of tincture, spirit, etc. As a solvent of essential oils and other odorous substances used in perfumery.

